# Distress and Resilience in Resettled Refugees of War: Implications for Screening

**DOI:** 10.3390/ijerph18031238

**Published:** 2021-01-30

**Authors:** Michael Hollifield, Eric C. Toolson, Sasha Verbillis-Kolp, Beth Farmer, Junko Yamazaki, Tsegaba Woldehaimanot, Annette Holland

**Affiliations:** 1 VA Long Beach Healthcare System, Long Beach, CA 90822, USA; 2 Department of Psychiatry and Behavioral Sciences, The George Washington University School of Medicine, Washington, DC 20052, USA; 3 War Survivors Institute, 5318 2nd Street, #703, Long Beach, CA, 90803, USA; 4 Department of Biology, The University of New Mexico, Albuquerque, NM 87131, USA; toolson@unm.edu; 5 Consultant, 3630 N. Winchell St., Portland, OR 97217, USA; sverbilliskolp@gmail.com; 6 Portland State University School of Social Work, Academic Student Recreation Center, Ste. 600, 1800 SW 6th Ave., Portland, OR 97201, USA; 7 International Rescue Committee, 1200 S. 192nd St., SeaTac, WA 98148, USA; Elizabeth.Farmer@rescue.org; 8 Asian Counseling and Referral Service, Seattle, WA 98144, USA; junkoy@acrs.org (J.Y.); tsegaba@u.washington.edu (T.W.); 9 Public Health Seattle & King County, Seattle, WA 98121, USA; Annette.Holland@kingcounty.gov

**Keywords:** health trajectory, refugee health, war, emotional distress, screening

## Abstract

There is little work published about predictors of specific trajectory types of distress in refugees of war during early resettlement in a host country. Data about distress (Refugee Health Screener—15 (RHS-15)) and possible predictors of distress were collected at the domestic medical examination (T1) within 90 days of arrival and the civil surgeon examination (T2) 11–16 months after T1 for refugee groups from three countries (COU). Descriptive, correlative, analyses of variance, and regression techniques were used to determine trajectory type and their predictors. A higher percentage (7.3%) were distressed at T2 than at T1. By group, the Bhutanese became more distressed, the Burmese became less distressed, and Iraqi’s continued to have high distress. A regression model showed gender, loss, post-migration stress, and self-efficacy to be significant predictors of trajectory type (R^2^ = 0.46). When the T1 RHS-15 score was added to the model, observed variance increased (R^2^ = 0.53) and T1 RHS score accounted for the majority of variance (*r* = 0.64, *p* < 0.001), with post-migration stress accounting for markedly less (β = 0.19, *p* = 0.03). Loss and self-efficacy became less significant. Loss was, however, a strong predictor of delayed and chronic distress trajectory type. These data suggest that screening for distress should occur at least twice during resettlement to detect those with initial distress and those with delayed distress. Screening should be coupled with identifying other social determinants of health and a comprehensive assessment to determine the need for intervention for secondary prevention (i.e., reducing delayed distress) and treatment (reducing chronic distress).

## 1. Introduction

Worldwide, seventy-nine million persons are forcibly displaced from their homes. Twenty-six million are refugees, 85% of whom are hosted in developing countries. From 1975 to 2017 more than three million refugees were invited to resettle in the United States [1]. Cross-sectional studies show that the majority of refugees experience multiple distressing symptoms, and a significant minority suffer from diagnostic-level psychiatric disorders [2,3,4,5,6,7,8,9,10], which are associated with stressful war- and migration-related events in a dose-dependent manner [7,11,12,13]. Furthermore, the overall health of refugees is compromised by the known association of chronic stress on health, in particular cardiovascular [14,15,16,17,18,19,20,21,22,23] and inflammatory [17,18,24,25,26] symptoms and disease [27]. Refugees experience significant health disparities over time due to an initially high health burden on arrival coupled with poor access to and engagement in general and mental health services [28]. Findings of Wong and colleagues epitomized this poor health trajectory over time by showing the unusually poor health status of Cambodian refugees two decades after resettlement in the U.S. [29].

Because incoming refugees have significant distress coupled with a high risk for poor overall health, screening for distress and referring for care during resettlement is warranted. Mental health screening at the domestic medical examination is supported by the Centers for Disease Control [30]. However, there are significant barriers to routine screening, including system and refugee factors [31] and a lack of knowledge about the clinical- and cost-effectiveness of screening and appropriate timing during resettlement. There are studies about distressing symptoms over time during the resettlement period that might inform screening protocols. Lie found that, over a 3-year post-resettlement period in Norway, anxiety and depression levels were unchanged, posttraumatic stress (PTSD) levels increased, and pre- and post-resettlement stressors were risk factors for worsening health status [32]. Ryan and colleagues showed similar levels of distress over one year in asylum-seekers in Ireland, mitigated by improved legal status [33]. Likewise, Steel and colleagues found similar rates over time, but rates were lower in asylees with permanent vs. temporary visas [34]. A longitudinal study in Sweden and Kosovo also showed a two-fold increase in PTSD symptom prevalence in refugees from Kosovo in an 18-month post-settlement period [35]. Another 3-year longitudinal study of Bosnian refugees determined that PTSD and depression symptoms persist during the resettlement period [36]. Kaltenbach and colleagues recently reported on a sample of refugees and asylum seekers from 7 regions, finding no change in distress or symptoms over a one-year period on a population basis and no change in the majority of individuals [37]. Comtesse recently showed that, 11 years after the first assessments, there was differential distress between those who stayed in Sarajevo throughout the war in Bosnia and Herzegovina versus former internally displaced persons and returnees who had sought refuge in other countries during the war [38].

There are no studies, however, about specific trajectory types of distress and their predictors over time in adult refugees of war. Muller identified four trajectory types (unremarkable, reacting, adapted, and persisting) on trauma and anxiety and depression symptoms in adolescent asylum seekers [39]. The authors found a general reduction of distress over time with the most common trajectory being “unremarkable” (no distress at either time) for anxiety and depression. Baseline symptom scores were the most robust single predictor for all mental health outcomes at follow-up

We present findings from the “Pathways to Wellness: Integrating Refugee Health and Well-being” project (P2W) about the prevalence and predictors of specific distress trajectory types of newly arrived refugees from three countries. Utilizing theoretical and empirical work about symptom trajectories in populations affected by war and mass causality [40], four trajectories between two time points during the first year of resettlement were evaluated. The *resistance trajectory* is one where individuals never acquire significant distress symptoms. The *resilience trajectory* is characterized by initial symptoms followed by recovery. The *chronic distress trajectory* is when individuals are initially symptomatic and remain so. The final trajectory, termed the *delayed distress trajectory*, is characterized by the lack of initial distress followed by later distress. Our hypotheses were that trajectories of distress: (1) will vary in prevalence by country of origin (COU) and (2) are affected by trauma severity, resource loss, post-migration stress, and self-efficacy. 

## 2. Materials and Methods

### 2.1. Design

A longitudinal design assessed distress at two time points: one was the domestic medical examination (DME) conducted within the first 90 days of arrival, the second was the civil surgeon examination (CSE) conducted between 11 and 16 months after the DME. Predictor factors (demographics, trauma, resource loss, post-migration stress, self-efficacy) were assessed at the DME.

### 2.2. Sample Frame, Sampling, Setting

The sample frame was all refugees age > 14 from three countries (Bhutan, Iraq, and Burma) speaking four languages (Nepali, Karen, Arabic, and Burmese (Karenni and Chin ethnic groups) at Public Health Seattle King County (PHSKC) for their domestic medical screening in Washington state. This sample frame was chosen as the most numerous refugee groups being resettled during the study period. Consecutive sampling of all eligible persons was conducted on pre-specified days by the P2W research coordinator at PHSKC during the DME between April 2010 and November 2010. All available refugees in this cohort were re-evaluated at the CSE between October 2010 and September 2011.

Ethical review and approvals were conducted by the Pacific Institute for Research and Evaluation and the ethics committee at Public Health Seattle and King County (PHSKC).

### 2.3. Procedures and Instruments

#### 2.3.1. Translation of Instruments

Translation is complex and must be adapted for specific purposes [41]. All instruments were translated using a rigorous, iterative back-and-forth participatory consensus process with refugees from each language group. This process ensured relevant language-specific semantics and cultural equivalence yielding accuracy and clarity of meaning across groups [13,42]. For the P2W study all scales were translated into: Burmese, Karen, Nepali and Arabic.

#### 2.3.2. Procedures

Written informed consent was obtained and distress assessment conducted by the P2W research coordinator with trained interpreters at the DME. Diagnostic proxy (DP) and predictor instruments were administered by the coordinator within two weeks of the DME. Health staff at PHSKC assessed distress during the CSE, and a second P2W coordinator with interpreters administered DP instruments within 3 weeks of the CSE. Refugees were given the choice of self-administration with instructions or of interview administration to parallel clinical procedures at PHSKC. 

#### 2.3.3. Distress: The Refugee Health Screener—15 (RHS-15)

The RHS-15 has 13 symptom items, one coping item, and one distress thermometer (DT). The instructions for the symptom items are to “indicate the degree to which the symptom has been bothersome to you over the past month.” Possible responses are: 0 = not at all, 1 = a little bit, 2 = moderately, 3 = quite a bit, and 4 = extremely. To help address variable literacy and cultural norms for understanding scale formats, there are symbols of jars with beans in them over the possible responses, with variable amounts of beans relevant to each scale response. The coping item assesses one’s beliefs about their general ability to cope with stress, and responses ranges from 0 (able to handle or cope with anything that comes your way) to 4 (unable to handle or cope with anything). The 14 symptom and coping responses are added to obtain a “total score.” The DT looks like a thermometer, with a “0” (“no distress—things are good”) at the bottom and a “10” (extreme distress—I feel as bad as I ever have”) at the top. A “RHS-15 positive case” is defined as a total score of ≥12 or a DT ≥ 5. The RHS-15 has shown validity to DP’s in the three refugee groups sampled [43,44]. Follow-up research has confirmed the validity of the RHS-15 and a shorter RHS-13 in multiple refugee groups and contexts [44,45,46,47].

The RHS-15 was in development during the first stage of this study. The distress score at the DME is from an interpolated version of the RHS-15 as previously described [43]. The distress score at the CSE is from the final compatible RHS-15 version.

#### 2.3.4. Diagnostic Proxy Instruments

Very few instruments that assess symptoms as DP’s have been developed for refugees [48]. None are definitive diagnostic equivalents. The Hopkins Symptom Checklist-25 (HSCL-25) is a valid indicator of anxiety and depression for the general U.S. population and for Indochinese refugees [48,49,50,51] and demonstrates transcultural validity [52,53]. Item-average scores ≥1.75 predict clinically significant anxiety (ANX) and depression (DEP) on the respective scales in general U.S. and refugee samples and are considered valid DP’s [49,51].

The Posttraumatic Symptom Scale-Self Report (PSS-SR) predicts PTSD diagnosis in U.S. populations [54]. Cronbach alpha is 0.91, and one-month test-retest reliability is 0.74. The 17 items on the scale, each scored from 0 to 3 for symptom frequency, are DSM-IV PTSD diagnostic items. The PSS-SR may be scored as continuous or a dichotomous DP. PSS-SR continuous scores and the DP are highly correlated with war-related trauma and impairment in Kurdish and Vietnamese refugees [13], and Cronbach alpha in these samples was 0.95.

#### 2.3.5. Predictor Instruments

War-related trauma was assessed with the Comprehensive Trauma Inventory-12 (CTI-12), which was adapted for this study from the Comprehensive Trauma Inventory-104 (CTI-104), a self-report instrument that reliably assesses a broad range of war-related events experienced by Kurdish and Vietnamese refugees and is a valid predictor of adverse mental health outcomes [13,55]. The CTI-12 consists of the 12 items that showed the strongest association with adverse mental health. Each item has 5 possible responses to reflect number of occurrences of each event: 1 = “*none*,” 2 = “*one or two times*,” 3 = “*three to twelve times*,” 4 = “*thirteen to fifty times*,” and 5 = “>50 times.” Scoring may be either a sum of dichotomous values (number of events positive) or a sum of occurrences, which was used for this study. Cronbach’s alpha for the current sample was 0.83.

Resource Loss (and gains) was assessed with 31 items deemed most relevant from refugee informants from the Conservation of Resources—Evaluation (COR-E) in five domains: personal, interpersonal, material, work, and health [56]. The COR-E loss scales have been shown to be predictors of distress and PTSD in Israeli’s exposed to terrorism and in American respondents after the World Trade Center attacks on 9/11 [57,58,59]. Respondents rated their extent of loss on a 0 to 4-point scale with the aid of a likert-type bar as guide. Item responses were summed so that higher scores reflected more loss. Cronbach’s alpha is not appropriate for these items since one type of loss is not necessarily related to others.

Post-migration stress was assessed with The Post-migration Living Problems (PMLP) Scale [60,61,62]. The 23 items are each rated on a 5-point scale from “no problem” to “a very serious problem” with the total score being a sum of item scores. The PMLP has 5 principal components accounting for 70% of the variance: (1) refugee determination process; (2) health, welfare and asylum problems; (3) family concerns; (4) general adaptation stressors; and (5) social and cultural isolation. Confirmatory factor analysis with our data supported the validity of these indicators.

Self-efficacy was assessed with the 10-item General Self-Efficacy Scale. (e.g., “I am confident that I could deal efficiently with unexpected events’’) [63]. Respondents indicated how true each statement was on a 4-point scale. Summed responses yielded a total score, with higher scores reflecting higher levels of self-efficacy. Cronbach’s alpha in the current sample was 0.90. 

### 2.4. Data Analyses

#### 2.4.1. Definitions

Distress was defined as a positive RHS-15 case. Time 1 (T1) was at the DME, and Time 2 (T2) at the CSE. Trajectory was defined as distress status at T2 relative to T1; see introduction for detail. The number and percent with each of the 4 trajectories by COU and gender was determined by frequency analyses.

#### 2.4.2. Trajectory Predictors

Logistic regression and analyses of variance (ANOVA) were utilized to test the hypotheses that distress trajectory will vary by COU and are affected by trauma severity, resource loss, post-migration stress, and self-efficacy. Logistic regression analyses of trajectory by COU and gender were conducted using Proc Logistic (SAS v. 9.3, SAS, Cary, NC, USA). Trajectory categories (Resistant, Resilient, Delayed, and Chronic) were treated as nominal variables and analyses were therefore based on the generalized logit model. Distress at T2 was treated as an ordinal variable with “distress” = 1 and considered ‘worse’ than “non-distress = 0, allowing use of proportional odds model. Analyses were conducted on separate and pooled data for each COU group using stepwise logistic regression with the Bayesian Information Criterion (Schwarz Criterion in SAS) being used to select the best model [64]. ANOVA was utilized to evaluate trajectory predictors by each COU and by each trajectory course.

## 3. Results 

### 3.1. Description of the Sample

**Figure 1:**
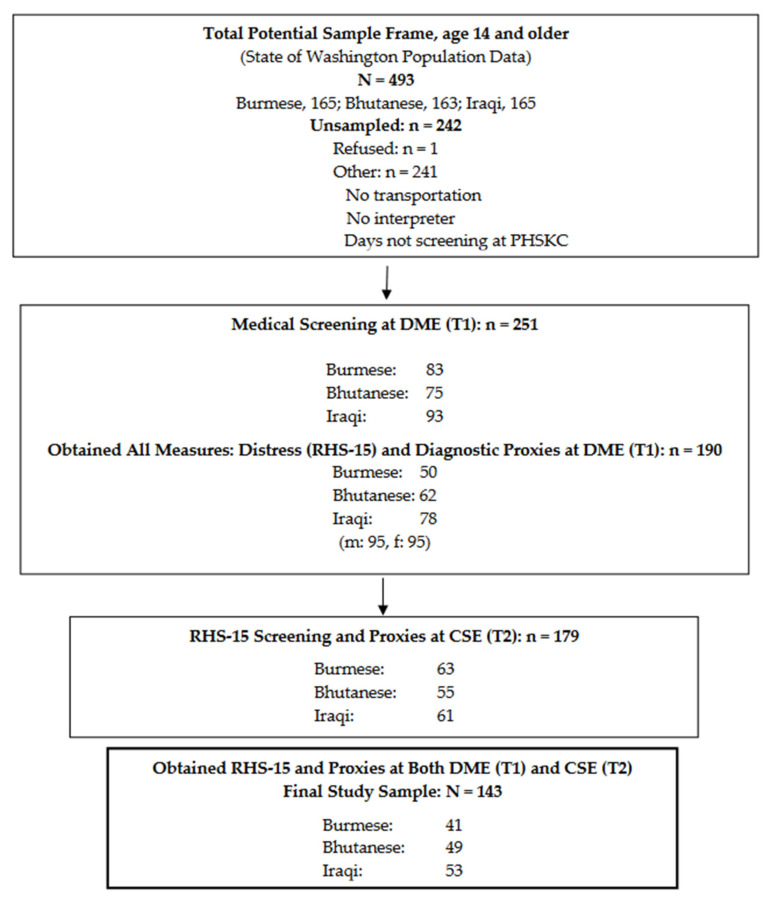
Sample Frame, Sample, and Subject Flow.

Figure 1 shows the sample frame, sample, and flow. During the study period PHSKC there were 493 potential participants, of which 251 were sampled at T1, and 190 were administered the diagnostic proxies (RR 76%). Of the 251, 179 returned for evaluation at T2, and of those, 143 had been evaluated with the RHS-15 at both T1 and T2, consisting of 49 Bhutanese (24 female, 25 male), 53 Iraqi’s (29 female, 24 male), and 41 Burmese (18 female and 23 male). Of the 251 sampled at T1, 77 were referred for mental health treatment, 57 accepted treatment, and 48 engaged in any treatment. Treatment outcome data were not collected. There were no clear COU differences by treatment engagement.

### 3.2. Distress and Trajectories by Country of Origin

In this sample a higher number (percentage) of refugees (12; 7.3%) were distressed at T2 than at T1. Table 1 shows that COU groups differ on distress at both time points (Fisher’s Exact at T1, Pr ≤ P = 0.004; at T2, Pr ≤ P = 1.05 × 10^5^ and that on a population basis, relative to T1, at T2 the Bhutanese became more distressed, the Burmese became less distressed, and Iraqi’s continued to have similar high rates of distress. Gender within COU group did not differ significantly on distress at either time point or on trajectory type, allowing for pooled analyses. 

Table 2 shows that the 4 trajectory patterns did not differ by gender within COU group (Fisher’s Exact Pr ≤ P, Bhutan 0.52, Iraq 0.78, Burma 0.12) and did differ by COU group (Fisher’s Exact Pr ≤ P, 1.24 × 10^6^. The Burmese showed the most resistance (68.3% compared to 35.8% of Iraqi’s and 36.7% of Bhutanese) and resilience (14.6% compared to 7.5% of Iraqi’s and 0% of Bhutanese). The Bhutanese showed the most delayed distress (30.6% compared to 7.5% of Iraqi’s and 7.3% of Burmese), and the Iraqi’s reported the most chronic distress (49.1% compared to 32.7% of Bhutanese and 9.8% of Burmese).

Distress on the RHS-15 generally paralleled that of the diagnostic proxies. Pearson’s bivariate correlation between T1 RHS-15 total score and PTSD, Anxiety, and Depression scores were 0.90, 0.87, and 0.87, respectively and between T2 RHS-15 total score and PTSD, Anxiety, and Depression were 0.89, 0.91, and 0.82, respectively. Table 3 shows the number and group percentage of the 3 diagnostic proxies at both times.

### 3.3. Impact of Trauma Severity, Resource Loss, Post-Migration Stress, and Self-Efficacy on Trajectory

Table 4 shows main effect differences by COU on all predictor variables. Burmese and Iraqis experienced significantly more war-related trauma (“trauma”) than the Bhutanese, who experienced significantly more resource loss (“loss”) than their counterparts. Bhutanese and Iraqis experienced similar levels of post-migration stress (“PMstress”) and significantly more than the Burmese. Bhutanese rated themselves with higher levels of self-efficacy (“SE”) compared with both Iraqis and Burmese, and Iraqis had higher levels than Burmese. A striking finding was that, in spite of less trauma and relatively high SE, the Bhutanese who showed moderate levels of distress at T1 and had the most delayed distress also had the most loss and significant PMstress.

Table 5 depicts the effects of predictor variables on trajectory assessed by ANOVA. War trauma is a stronger predictor of chronic distress and resilience compared to delayed distress. Loss is a highly significant predictor of both chronic and delayed distress. PMstress better predicts that chronic distress will be the most likely trajectory than either delayed distress or resistance, and delayed distress as more likely than resistance. Counter-intuitively, higher SE best predicts a delayed distress trajectory over either a resilient or chronic distress trajectory. These data highlight the significant associations between fewer losses with resistance and resilience, and less PMstress with resistance.

Table 6 shows three regression models of predictor variables on trajectory. Each model was significant and a good fit for the data. Predictor variables trauma, loss, PMstress, and SE were entered into the first regression model using the forced method to ensure replicability. The first regression model produced R^2^ = 0.42, F (4, 114) = 20.92, *p* < 0001, where loss (β = 0.44, *p* < 0.001), PMstress (β = 0.27, *p* = 0.004), and SE (β = −0.15, *p* < 0.05) all contributed differentially to the model. Loss has the greatest effect on trajectory. 

When the variables gender and COU were added to the regression model, a slight increase in variance was observed (R^2^ = 0.46, F (6, 112) = 15.71, *p* < 0001). The associations between gender-Trajectory and COU-Trajectory were both weak relationships, and the added variance was small though significant for gender (β = 0.15, *p* = 0.037) but not for COU (β = 0.15, *p* = 0.112). Loss (β = 0.35, *p* = 0.001), PMstress (β = 0.28, *p* = 0.002), and SE (β = −0.17, *p* = 0.025) remained significant after the addition of gender and COU to the model.

In the third regression model, T1 RHS Score was added to the model and increased observed variance (R^2^ = 0.53, F (7, 111) = 17.61, *p* < 001). T1 RHS score was significantly associated with Trajectory (*r* = 0.64, *p* < 0.001). PMstress accounted for markedly less yet still significant variance (β = 0.19, *p* = 0.03), and loss (β = 0.12, *p* = 0.27) and SE (β = −0.06, *p* = 0.45) were no longer significant. 

**Figure 2:**
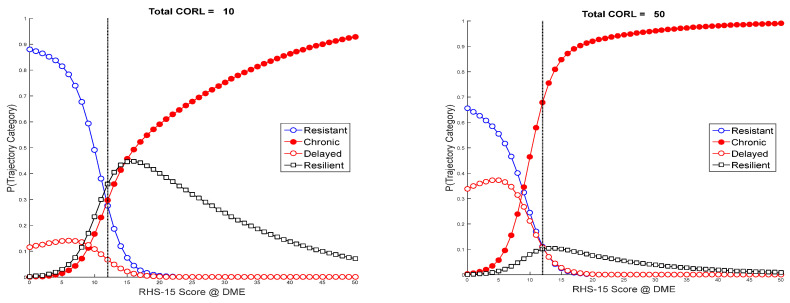
Effect of loss score on trajectory probability.

Since loss was a strong predictor of delayed and chronic distress, probability analyses were conducted to show interactive effects of loss by trajectory type. Figure 2 shows that, with a low loss score (e.g., 10), the likelihood of resistance, resilience, delayed distress, and chronic distress at an RHS cut score of 12 at T1 is 0.28, 0.35, 0.07, and 0.30, respectively, while with a high loss score (i.e., 50), probabilities are 0.11, 0.10, 0.11, and 0.68, respectively.

## 4. Discussion

The first hypothesis, that distress trajectory type during early resettlement varied by COU, was confirmed. Trajectory type did not vary by gender. The Burmese showed the highest prevalence of resistance (68%) and resilience (15%), in addition to relatively low delayed (7%) and chronic (10%) distress. Seventy-six percent of Burmese were not distressed on arrival and of the relatively low 10 persons (24%) that were distressed on arrival only 3 (7% of total Burmese sample and 30% of the initially distressed) remained distressed at the second assessment. On the other hand, six persons (15% of the Burmese sample and 60% of initially distressed) became non-distressed by the second visit. Nepali Bhutanese showed different patterns: there was a 33% prevalence of distress on arrival, none became non-distressed by the CME, and 15 of 31 persons (31% of the Bhutanese sample and 45% of initially non-distressed) became distressed, resulting in 64% being distressed at the CSE. The Bhutanese thus had the highest prevalence of delayed distress among the three groups. Iraqi’s had the greatest initial rates of distress (57%) which remained stable on a population basis from DME to CSE: 7.5% of those distressed became non-distressed, and 7.5% of initially non-distressed persons became distressed. Iraqi’s thus showed the highest prevalence of chronic distress. 

A few critical themes emerge from these results. First, the large minority of newly-resettled refugees in all three COU groups are resistant to the severe stress experienced during war, oppression, and migration. The majority (60%) were not significantly distressed at the initial examination, and the most likely trajectory was resistant (45%). The second theme was that distress from T1 to T2 was stable. Those who were initially distressed were not likely to get better in the initial resettlement period: across all groups only 8% followed the resilient trajectory. The second and third most likely trajectories were chronic (32%) and delayed (15%) distress respectively. Stability over time, both resistance and chronic distress, was thus most likely and was primarily a function of distress score at the initial domestic medical examination. Change, both resilience and delayed distress was less common and primarily a function of country of origin. In all, 22 (15%) refugees experienced delayed distress, 15 of whom were Bhutanese, while 10 (7%) experienced resilience, six of whom were Burmese. It is not clear from our data if treatment engagement had any impact on trajectory type since only 19% of the sample at T1 engaged in treatment and outcome data were not available.

The second hypothesis, that trauma severity, post-migration stress, resource loss, and self-efficacy would have some predictive capacity for, or explain some variance in distress trajectory experienced was also supported in part. ANOVA with post-hoc pair analyses showed that some of these four predictor variables differed between country groups, which in turn had some predictive capacity for distress trajectory. Burmese and Iraqi’s suffered more war-trauma (by the CTI-12) than Bhutanese. Bhutanese reported more loss than Iraqi’s, and both of these groups reported more loss and post-migration stress and endorsed higher self-efficacy than Burmese. Those experiencing the resilient trajectory had significantly more war-trauma than those on the resistant or delayed distress trajectory, and those with chronic distress had more trauma than those with delayed distress. Put another way, those with initial distress tended to have experienced more war trauma than those who were not initially distressed. Refugees with chronic distress reported more resource loss than refugees on the other three trajectories, and those with delayed distress had more loss than those on the resistant trajectory. The same basic pattern was true for post-migration stress, except that those on the resilient trajectory tended to have lower post-migration stress than those with chronic distress, though this was not statistically significant. Delayed distress was associated with higher initial self-efficacy compared to those resilient or with chronic distress. One interpretation is that perceived self-efficacy at the initial evaluation protects against reporting of initial distress, but other factors such as post-migration stress are more powerful in creating distress over time. This may help explain what has been termed the “honeymoon” period [47], where refugees report a sense of wellbeing during early resettlement due to being in a new country with perceived new opportunities, but slowly lose the sense of wellbeing as exposure to a myriad of post-migration stressors and hardships increase. While trauma severity, post-migration stress, resource loss, and self-efficacy had some predictive capacity on trajectory, initial distress was, however, overwhelmingly the best predictor of trajectory.

While this is the first study we know of that examines factors associated with specific distress trajectory types in adult refugees of war, these data are supported by other work evaluating effects of both pre- and post-migration factors on distress. Earlier studies showed that post-migration stress in addition to war-trauma contributes to poor mental health in refugees [60,65,66]. Research in the past decade has verified that demographic and post-migration variables influence refugee distress [3,6,9,10,67,68,69]. A number of studies evaluating the relative contribution of types of stressors have found that post-migration stress often provides a risk for distress similar to or greater than war-related trauma [3,7,9,61,70,71,72]. Pre- and post-migration stress may differentially predict specific kinds of symptoms and distress in both children and adults [73,74]. Lost resources and ongoing stressors have been consistently found to predict the risk for developing and maintaining a stress-related illness following trauma in war survivors, as well as in survivors of natural disasters, terrorist attacks, intimate partner violence, and sexual assault [40,59,75,76,77,78,79,80,81]. Recent work has deepened knowledge about variables associated with refugee distress, such as gender, age, religion practiced, receipt of medical services, instability of housing [82], negative life events and employment status [37]. With recognition of the contributions of loss and ongoing stress to the health of war survivors, investigators are pursuing a broader social ecological model of trauma and recovery [83]. To determine how this knowledge may be utilized to encourage trauma recovery, Betancourt and colleagues conducted focus groups with Somali refugee adolescents, mothers, and fathers to identify five forms of resources—religious faith, healthy family communication, support networks, peer support, and community talk—that may have been compromised during war and flight—that are potentially protective if restored and can help to offset acculturative and resettlement stressors [84].

The growing evidence for effective interventions for refugees [85,86,87,88,89,90,91,92,93,94], coupled with the significant prevalence of distress and attendant risks of ongoing morbidity and mortality, argues for universal screening for newly resettled refugees. The current study data suggest that screening should occur at least twice in the course of resettlement to detect those with initial distress and the significant number of those with delayed distress. Screening should, of course, be coupled with identifying other social determinants of health and a comprehensive assessment to determine the need for intervention for secondary prevention (i.e., reducing delayed distress) and treatment (reducing chronic distress). Other investigators have also found a significant prevalence of chronic distress to support this conclusion [37,68]. What is not clear from this or other research is: (1) the best timing of the second screening, (2) who may not need a second screening, and (3) the public health benefit of screening on health outcomes. Until more data are available to clarify these questions, screening should be conducted in a reliable manner by personnel who are trained to administer and properly interpret and act on screening information. Similar to screening for any other health condition, screening for emotional distress and psychiatric disorder is distinct from diagnostic assessment or treatment planning, which needs to be conducted by persons skilled in these tasks.

Study limitations include a sample with only three groups from three countries, inclusion of a limited number of predictor variables, high variability of scores on predictor variables, and inability to account for concurrent treatment. Thus, conclusions about the generalizability of these data to other COU groups is not possible, all factors accounting for distress are not identified, and predictions about distress trajectory for individuals is less reliable than for groups and is not informed by concurrent treatment. Complex multivariate modeling utilizing more predictor variables with a larger sample from different countries and contexts may allow for more definitive conclusions about the effects of contextual variables on distress/non-distress trajectories in newly resettled refugees. Constructs of distress and trajectory are narrowly defined, where broader construct definitions may produce different results. Defining and assessing each construct using scales developed from Western education can also present challenges of validity, particularly for less literate participants. In spite of these limitations, the four hypothesized predictor variables explained 42% of the trajectory type variance, and with the addition of country of origin, gender and initial distress, the model accounted for 53% of the variance. 

## 5. Conclusions

The current study adds to the body of knowledge by estimating the prevalence of specific distress trajectory types and identifying important variables that account for 53% of the variance of trajectory type in adult refugees of war after resettlement in the U.S. It also further reifies literature that favors early intervention by screening refugees for emotional distress at least twice during resettlement and supports our contention that assessing loss and post-migration stress will augment extant clinical information about who is likely to stay distressed, become distressed, or be resilient. 

## Figures and Tables

**Table 1:** RHS-15 case status (Positive = above RHS-15 cutscore; Negative = below RHS-15 cutscore) by country at DME (T1) and CSE (T2).

	Country of Origin		
	Bhutan (*n* = 49)	Iraq (*n* = 53)	Burma (*n* = 41)
DME Negative	33	23	31
DME Positive	16 (32.6%)	30 (56.6%)	10 (24.4%)
CSE Negative	18	23	34
CSE Positive	31 (63.3%)	30 (56.6%)	7 (17.1%)

**Table 2:** Distress trajectory by country of origin and gender.

		DME Negative (*n* = 87)		DME Positive (*n* = 56)	
		CSE Negative	CSE Positive	CSE Negative	CSE Positive
		Resistant (*n* = 65)	Delayed Distress (*n* = 22)	Resilient (*n* = 10)	Chronic Distress (*n* = 46)
Bhutan (*n* = 49)	Female	7	9	0	8
	Male	11	6	0	8
Iraq (*n* = 53)	Female	9	3	2	15
	Male	10	1	2	11
Burma (*n* = 41)	Female	10	3	2	3
	Male	18	0	4	1
Total	Female	26	15	4	26
Total	Male	39	7	6	20
Total	All	65	22	10	46

Resistant means RHS-15 negative at both DME (T1) and CSE (T2), Delayed Distress means RHS-15 negative at DME and positive at CSE, Resilient means RHS-15 positive at DME and negative at CSE, Chronic Distress means RHS-15 positive at both DME and CSE.

**Table 3:** Diagnostic proxy number (%) by country of origin at DME (T1) and CSE (T2).

	Bhutan *N* = 49		Iraq *N* = 53		Burma *N* = 41	
	DME	CSE	DME	CSE	DME	CSE
**PTSD**	10 (20.4)	21 (42.9)	26 (49.1)	32 (60.4)	6 (14.6)	5 (12.2)
**Anxiety**	9 (18.4)	17 (34.7)	20 (37.8)	24 (45.3)	4 (9.8)	0 (0)
**Depression**	9 (18.4)	21 (42.9)	20 (37.8)	24 (45.3)	4 (9.8)	0 (0)

**Table 4:** War Trauma, Loss, Post-migration stress, and self-efficacy by COU, M (SD).

	Bhutan (1)	Iraq (2)	Burma (3)	Total Sample	Statistics
**War-trauma** ***N* = 133**	16.4 (3.7)	20.5 (7.6)	21.2 (5.9)	19.4 (6.4)	F = 8.01; *p* = 0.001 3 > 1; 2 > 1
**Resource Loss** ***N* = 139**	50.9 (21.5)	35.0 (24.0)	19.6 (17.8)	36.4 (24.7)	F = 22.67; *p* < 0.001 1 > 2; 1 > 3; 2 > 3
**PMStress** ***N* = 127**	33.5 (13.6)	34.2 (18.7)	25.6 (15.1)	31.8 (16.5)	F = 3.11; *p* = 0.048 1 > 3; 2 > 3
**Self-Efficacy** ***N* = 137**	31.6 (6.3)	28.8 (7.0)	25.4 (6.0)	28.9 (6.9)	F = 9.40; *p* < 0.001 1 > 2; 1 > 3; 2 > 3

War trauma score is a sum of occurrences on the CTI-12; higher = more war trauma, Resource loss score is a sum of item severity score; higher = more loss, PM Stress score is the sum of item severity scores; higher = more PM stress, Self-efficacy score is the sum of item scores; higher = more self-efficacy.

**Table 5:** ANOVA of predictor variables on trajectory.

	Resistant (1)	Resilient (2)	Delayed Distress (3)	Chronic Distress (4)	Total	Statistics *
**War-trauma**	18.5 (5.6)	22.7 (4.8)	15.8 (3.4)	21.6 (7.6)	19.4 (6.4)	F = 5.8; *p* = 0.001
***N* = 133**	R = 12–40	R = 15–33	R = 12–27	R = 12–44	R = 12–44	2 > 1; 2 > 3; 4 > 3
**Resource Loss**	24.8 (20.5)	23.6 (16.9)	38.7 (20.3)	54.0 (23.1)	36.4 (24.7)	F = 17.9; *p* < 0.001
***N* = 139**	R = 0–75	R = 7–65	R = 2–71	R = 13–95	R = 0–95	4 > 1, 2, 3; 3 > 1
**PMStress**	22.4 (13.5)	33.5 (17.5)	31.7 (14.1)	43.4 (13.5)	31.8 (16.5)	F = 18.4; *p* < 0.001
***N* = 127**	R = 0–59	R = 16–67	R = 4–63	R = 16–68	R = 0–68	4 > 1, 3; 3 > 1
**Self-Efficacy**	29.5 (6.9)	25.1 (7.0)	32.3 (5.1)	27.1 (6.9)	28.9 (6.9)	F = 4.2; *p* = 0.007
***N* = 137**	R = 15–40	R = 11–36	R = 22–40	R = 8–38	R = 8–40	3 > 2, 4

Values are Mean (SD) for each predictor variable, R = range of score for predictor variable by trajectory type, * Multiple comparisons with Bonferroni correction, group difference at *p* < 0.05.

**Table 6:** Three regression models of predictor variables on trajectory, β, (*p* value).

Model	Loss	PMstress	Trauma	SE	COU	Gender	RHS DME	Stats
**1**	0.44 (<0.01)	0.27 (<0.01)	NS	−0.15 (0.05)	---	---	---	R^2^ = 0.42 F = 20.9
**2**	0.35 (<0.01)	0.28 (<0.01)	NS	−0.17 (0.03)	NS	0.15 (0.04)	---	R^2^ = 0.46 F = 15.7
**3**	NS	0.19 (0.03)	NS	NS	NS	NS	0.64 (<0.01)	R^2^ = 0.53 F = 17.6

## Data Availability

The data presented in this study are available on request from the corresponding author. The data are not publicly available due to initial informed consent agreements with this vulnerable population.
